# Editorial: Bone Marrow Adiposity: Establishing Harmonized, Mechanistic, and Multidisciplinary Approaches to Reach Clinical Translation

**DOI:** 10.3389/fendo.2020.604110

**Published:** 2020-10-27

**Authors:** Stephanie Lucas, Clifford James Rosen, Nathalie Bravenboer

**Affiliations:** ^1^Marrow Adiposity and Bone Lab-MABLab ULR4490, Univ. Littoral Côte d’Opale, Boulogne-sur-Mer, Univ. Lille, CHU Lille, Lille, France; ^2^Center for Clinical & Translational Research, Maine Medical Center Research Institute, Scarborough, ME, United States; ^3^Department Clinical Chemistry, Amsterdam Movement Sciences, Amsterdam University Medical Center, Amsterdam, Netherlands

**Keywords:** Bone Marrow Adiposity Society, bone marrow adipocytes, special issue, methodology, nomenclature

This Research Topic comprises ten articles describing different approaches to study the regulation or maturation of bone marrow adiposity. The conversion of the “red” hematopoietic bone marrow to a “yellow” fatty one is a long-known phenomenon that has been referred to by many names. Bone Marrow Adipocytes (BMAds) have also been revealed as puzzling yet intriguing cells, which could provide exciting novel insights into the pathophysiology and treatment of aging, osteoporosis, anorexia nervosa, obesity and diabetes, aplastic anemia, multiple myeloma, leukemia, bone metastases, and many other clinical conditions. Thus, it is no wonder that enthusiasm surrounding Bone Marrow Adiposity (BMA) research has accelerated in recent years, especially since techniques for the isolation and study of BMAds have become increasingly refined and established (**Figure 1**). However, literature on the role of BMAds shows considerable variation and inconsistencies so far. The need for consensus and uniformity in this growing field of research is therefore a major issue that has been highlighted by the International Bone Marrow Adiposity Society (BMAS).

**Figure 1:**
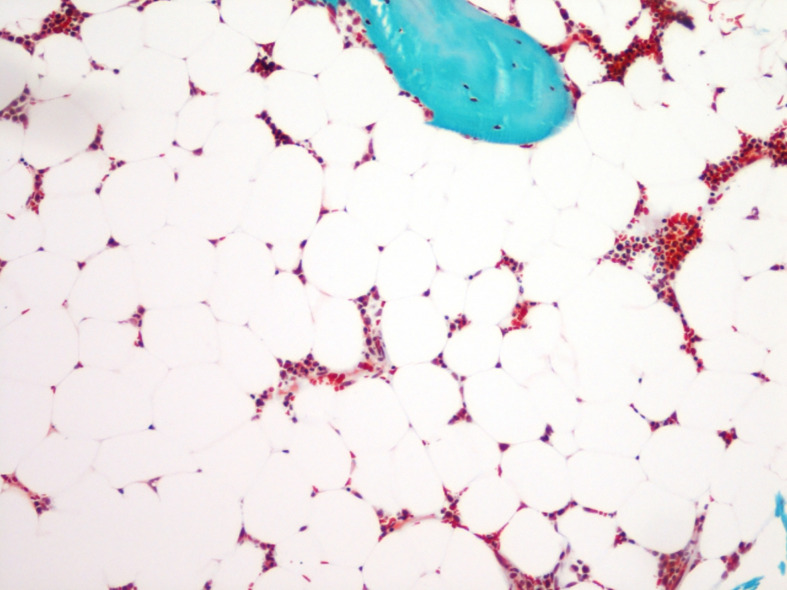
Bone marrow adiposity in a human transiliac bone biopsy. Bone marrow adipocytes appear as “gost” cells in a Goldner’s stained section. Magnification 10 × 10.

BMAS was founded in 2017 to consolidate the growing scientific community interested in BMA, after the success of meetings organized since 2015. This young society brings together physicians and scientists working on rheumatology and bone biology, oncology, hematology, medical imaging, endocrinology, and metabolic perturbations, for which BMA research provides a new scientific direction. So far there have been five meetings with an excellent standard of keynote lectures and research presentations, in combination with interactive discussions in working groups. Reports from the third and the fourth meetings are presented in this Research Topic (Corsi et al.; Penel et al.). BMAS is an open society with many investigators from diverse fields who have been gathering in working groups to share protocols and experience. Two of those working groups have important publications in this Research Topic (Bravenboer et al.; Tratwal et al.), which are sure to provide a foundation with a high impact on BMA research in the future. Other BMAS working groups are now finalizing additional position papers (*e.g.* for Biobanking), which will provide further important resources for future BMA research.

One position paper addresses the nomenclature challenge regarding the diverse terminology used so far to describe the fat depot, the adipose cells, their subtypes or their stem and progenitor cells within the bone marrow, as well as the methods used for their study (Bravenboer et al.). Indeed, BMA can be measured through histomorphometry, computed tomography (CT), and Magnetic Resonance Imaging (MRI), a diversity of techniques that has also led to a confusing heterogeneity. Recommendations of specific terms, abbreviations, and units have thus been discussed and compiled to propose a standardized nomenclature to be adopted by the BMA research community. As observed in the field of bone histomorphometry following the first consensus publication in 1987 (1) such terminological harmonization is expected to improve the consistency between studies and to facilitate collaborations and inputs from the diverse fields.

Another critical need related to methodological standards has also led to a second position paper (Tratwal et al.). This paper already has received many readings and downloads online, demonstrating that uniformity in methods has become indispensable to boost BMA research not only in quantity but also in quality. Based on the review of the literature and on expert opinions, specific gold-standard methodologies are discussed regarding 1) histomorphometry of BMAds, 2) *ex vivo* BMA imaging using *µ*CT following the staining with osmium tetroxide or other contrast-enhancing agents, 3) *in vivo* BMA imaging using MRI techniques in clinical studies, 4) cell isolation, culture, differentiation, and *in vitro* modulation of primary BMAd and BM stromal cell precursors, 5) lineage tracing and *in vivo* BMA modulation, and 6) BMA biobanking. Importantly, this second publication highlights the requirements for consensual annotations and standardization in techniques (choice of referent and condition) and provides guidelines for a detailed description of critical experimental parameters to minimize variability and to improve the comparability between studies. Emerging techniques are also described, which may soon come to complement or substitute the gold standards. One of these techniques is histomorphometry by unbiased semi-automatic image analysis; this is thoroughly described in this Research Topic by Tratwal et al. in an original research paper on MarrowQuant, a new image analysis plug-in. Other relevant technical strategies have also been developed and are described in the other associated articles.

Many scientists aimed to answer the urgent questions on how BMA is regulated. Several papers in this Research Topic address this subject. A well-known stimulus for BMA accretion is irradiation. It is therefore highly relevant that Costa and Reagan put into perspectives the consequences of therapeutic irradiation on Skeletal Stem Cell (SSC) properties and BMAT development in rodents and patients with skeletal complications (Costa and Reagan). In doing so, they propose new strategies in conjunction with radiotherapy. The central nervous system is a key mediator of adipose tissue function through sympathetic adrenergic neurons. Wee et al. investigate whether central autonomic pathways are also involved in BMAT regulation using viral transneuronal tract tracing in two mouse strains (Wee et al.). After quantifying the local sympathetic adrenergic innervation, they establish that BMAT shares common central neuroanatomic pathways, notably with peripheral adipose tissue, which paves the way for future studies on BMAT functional regulation. An interesting additional hypothesis is that temperature also regulates BMAT through the sympathetic nervous system. Turner et al. report the effects of propranolol in mice housed at 22°C or thermoneutrality to highlight that *β*-adrenergic receptor signaling contributes to regulating BMAT levels and metabolism without critically impacting on the premature cancellous bone loss observed at room temperature (Turner et al.). Chronic hyperglycemia has also been revealed as a promoting factor for adipogenesis within the bone marrow. Rharass and Lucas further investigate the impact of low and high glucose levels on human SSC-derived BMAds and their non-lipid-laden-cell counterparts (Rharass and Lucas). They demonstrate that high glucose concentrations drive only mature BMAds toward an altered phenotype through a rise in reactive oxygen species (ROS) generation. Finally, Dalla Valle et al. evaluate in this Research Topic the handling of free fatty acid (FFA) desaturation in human SSCs to prevent saturated FFA-induced lipotoxicity (Dalla Valle et al.). Using pharmacological agents to modulate LXR activity, they provide evidence for a protective role of the desaturase enzyme Stearoyl-CoA 9-Desaturase (SCD)1 in human SSC viability during saturated FFA exposure.

Most of the research in this Research Topic focuses on the regulation of BMAT and the differentiation routes of SSCs. Elucidation of these entangled pathways is crucial for targeting SSCs or BMAds for the treatment of osteoporosis or other metabolic (bone) diseases. For this ambition, we also need to clarify how BMAds regulate bone cells such as osteoblasts and osteoclasts. Unravelling the cross talk between BMAds and bone cells is crucial not only to target BMAds for improved bone health, but also if we are to uncover other mechanisms important in local and systemic metabolism.

## Author Contributions

NB and SL wrote the manuscript. CR corrected and approved the final manuscript. All authors contributed to the article and approved the submitted version.

## Conflict of Interest

The authors declare that the research was conducted in the absence of any commercial or financial relationships that could be construed as a potential conflict of interest.
